# The impact on undergraduate students

**DOI:** 10.7554/eLife.106705

**Published:** 2025-03-26

**Authors:** Reenie Tian, Kamna Kalkunte, Esha Pia, Gianna Dunn, Nina Bugg, Mayank Chugh

**Affiliations:** 1 ReForm Lab, College of William & Mary Williamsburg United States

**Keywords:** Science Under Threat in the United States, science, politics, careers in science, undergraduate students, early-career researchers, None

## Abstract

Anti-science policies, funding cuts, scientific censorship and the US withdrawing from international commitments are worrying members of the ReForm Lab at the College of William & Mary.

While researchers are bearing the brunt of the funding cuts being made by the Trump administration, undergraduate students will also be impacted. The authors of this article are five undergraduates and an early-career assistant professor (MC) at the College of William & Mary – a liberal arts institution where faculty balance research with teaching, providing mentored and foundational experiences to students – and we are worried that the anti-science policies of the current administration will adversely affect the future of biomedical research in the US, the workforce that sustains it, and the global communities it serves.

Cutting support for scientific progress isn’t just a setback for the research community; it is a deliberate attack on innovation, education and healthcare. With graduate admissions paused, acceptances rescinded, summer internships halted, federal datasets altered, and funding pulled from vital research programs, what do professors tell junior students who are women of color? What do professors tell the pre-med sophomore who dreams of a career in translational medicine and public health, but is now seeing funding for research being withdrawn? What do they tell the biology major who is contemplating a PhD, only to see even the most prestigious programs freeze applications due to financial and political pressures?

More pressingly, what do professors tell students about the state of science itself? That science is under attack – not just through funding cuts, but through ideological battles that question its very legitimacy and integrity? That political shifts threaten to undo decades of progress, leading to increased neglect of already vulnerable communities? How do we encourage students to stay in science when doing so might require them to suppress their beliefs, to disengage from politics, or to navigate a research landscape being shaped by forces beyond scientific expertise and community needs? Here are our personal perspectives.

**Reenie Tian:** I come from a community that trusts science – we get vaccinated, we drink pasteurized milk, and we don’t inject ourselves with hydroxychloroquine. Yet, I fear how anti-science rhetoric may fuel preventable outbreaks of diseases like measles and polio, causing needless suffering. In Texas, a child has died from measles for the first time in a decade. The US has forgotten COVID’s lessons and remains unprepared for future pandemics. My parents preached science as a secure career path, but as I apply to grad schools now, I worry about getting a spot – only to lose my funding or to get furloughed without notice. The rising tide of anti-science sentiment only deepens my concerns.

**Kamna Kalkunte:** My dream of a career in epidemiology and molecular biology feels increasingly out of reach. NIH-funded health research programs are shutting down, and students across the country, including my friends on campus, fear for their place in the biomedical workforce. I aspire to work for the World Health Organization (WHO), but with the US retreating from international agreements, I am unsure if the WHO will continue in its vital capacity. It is painful to watch years of scientific progress being dismantled by political leaders who are disconnected from the discipline.

**Esha Pia:** As a kinesiology major aspiring to enter medicine, I find the rise of anti-science policies deeply troubling. Medical school is already a daunting pursuit, but political decisions are making it even harder. Funding cuts threaten both current work and also future breakthroughs in patient care and public health. As a former intern at the Federal Emergency Management Agency (FEMA), I am alarmed by proposals to dismantle the agency, which plays a critical role in disaster response. The potential loss of FEMA would harm vulnerable communities and disrupt career paths for students like me who are dedicated to public service. It is disheartening to pour time into studying, volunteering and gaining clinical experience while watching the field I love become increasingly politicized and underfunded.

**Gianna Dunn:** The censorship of scientific writing is tainting my experience as an undergraduate researcher. I feel my freedom of speech eroding as federal scientists at the Centers for Disease Control (CDC) are instructed to withdraw articles that have been submitted to, or accepted by, journals because the articles do not align with the administration’s agenda. This threatens my future in global health. With the US withdrawing from the WHO, the Paris Agreement, and other international bodies and agreements, I worry about the ability of researchers in the US to make contributions to global health initiatives in the long term. As I consider graduate programs in public health, I feel increasingly anxious about how scientific censorship and funding restrictions will shape my field.

**Nina Bugg:** From fluoride’s role in preventing tooth decay to mapping the human brain, NIH funding has fueled biomedical breakthroughs that have helped the global community. It inspired me to pursue a degree in biology and later, I hope, a career in medical research to contribute to discoveries that could one day save lives. I worry about what anti-science policies mean – not just for me, but for patients waiting for new treatments and for communities relying on public health initiatives. For example, clinical trials among vulnerable communities in South Africa are being terminated before they finish because the administration is intent upon dismantling the US Agency for International Development (USAID). Without strong federal support, we risk losing a generation of researchers who might have developed the next breakthrough.

**Mayank Chugh:** I worry about my research, my teaching, my career and my ability to mentor students in an increasingly unstable environment. My lab focuses on how knowledge is created, shared, and governed at the intersection of biomedical science and social inequality – areas that are now under fire. Federal grant schemes that I had planned to submit applications to have been scrapped, and the prospect of censorship shaping my academic trajectory, tenure prospects and research direction is deeply unsettling. However, when I asked my students, where do we go from here?, their answer was reassuring: “We keep marching on.”

At a recent campus talk, the multidisciplinary artist Kamara Thomas said: “Let limitations be a collaborator.” That sentiment resonates deeply with us in today’s sociopolitical climate. Even as resources dwindle and opportunities shrink, we are determined to embrace interdisciplinary collaboration, foster transparent dialogue, and push forward with rigorous, openly accessible research and community building.

## Note

The authors are all writing in a personal capacity and the views expressed in this article do not represent the position of the College of William & Mary.

